# A novel interaction between ATOH8 and PPP3CB

**DOI:** 10.1007/s00418-015-1368-5

**Published:** 2015-10-26

**Authors:** Jingchen Chen, Ajeesh Balakrishnan-Renuka, Nina Hagemann, Carsten Theiss, Verena Chankiewitz, Jinzhong Chen, Qin Pu, Kai S. Erdmann, Beate Brand-Saberi

**Affiliations:** Department of Anatomy and Molecular Embryology, Medizinische Fakultät, Ruhr-Universität Bochum, Abt. f. Anatomie und Molekulare Embryologie, Geb. MA, 5/158, 44780 Bochum, Germany; Department of Neurology, University Hospital Essen, 45122 Essen, Germany; Department of Cytology, Ruhr-University Bochum, 44780 Bochum, Germany; Department of Genetics, Fudan University, Shanghai, People’s Republic of China; Department of Biomedical Science & Centre for Membrane Interactions and Dynamics (CMIAD), University of Sheffield, S10 2TN Sheffield, UK; Department of Craniofacial Development and Stem Cell Biology, King’s College London, SE19RT London, UK

**Keywords:** ATOH8, PPP3CB, Calcineurin, Cyclosporin A

## Abstract

ATOH8 is a bHLH transcription factor playing roles in a variety of developmental processes such as neurogenesis, differentiation of pancreatic precursor cells, development of kidney and muscle, and differentiation of endothelial cells. PPP3CB belongs to the catalytic subunit of the serine/threonine phosphatase, calcineurin, which can dephosphorylate its substrate proteins to regulate their physiological activities. In our study, we demonstrated that ATOH8 interacts with PPP3CB in vitro with different approaches. We show that the conserved catalytic domain of PPP3CB interacts with both the N-terminus and the bHLH domain of ATOH8. Although the interaction domain of PPP3CB is conserved among all isoforms of calcineurin A, ATOH8 selectively interacts with PPP3CB instead of PPP3CA, probably due to the unique proline-rich region present in the N-terminus of PPP3CB, which controls the specificity of its interaction partners. Furthermore, we show that inhibition of the interaction with calcineurin inhibitor, cyclosporin A (CsA), leads to the retention of ATOH8 to the cytoplasm, suggesting that the interaction renders nuclear localization of ATOH8 which may be critical to control its activity as transcription factor.

**Electronic supplementary material:**

The online version of this article (doi:10.1007/s00418-015-1368-5) contains supplementary material, which is available to authorized users.

## Introduction

ATOH8 is a basic helix-loop-helix (bHLH) transcription factor (Chen et al. 2011), involved in various developmental programs such as neurogenesis (Inoue et al. 2001; Yao et al. 2010), differentiation of pancreatic precursor cells (Lynn et al. 2008) podocytes (Ross et al. 2006), muscle development (Yao et al. 2010; Balakrishnan-Renuka et al. 2014), differentiation of endothelial cells (Fang et al. 2014) and muscle fiber regeneration (Guttsches et al. 2015). *Atoh8* has recently also been identified as a novel oncogene candidate as it shows abnormal DNA copy number in glioblastoma multiforme (Freire et al. 2008). In mouse, it is expressed widespread in various types of tissues and organs, suggesting its multiple roles in different cellular contexts (Wang et al. 2015). It is proposed that *Atoh8* expression may be regulated by tissue-specific bHLH transcription factors in different cellular contexts so that it can play exclusive roles in different developmental events (Pujadas et al. 2011). The viability of mice deficient in this gene depends on the knockout regions ranging from early embryonic death (Lynn et al. 2008) to survival into adulthood (Rawnsley et al. 2013). Recent studies start revealing the molecular mechanism how ATOH8 regulates different developmental events: In pancreatic mPAC cells, ATOH8 acts as a Neurogenin repressor and its repression activity could probably be attributed to its binding to E47 protein, which may inhibit the activity of E47/E47 or E47/Neurogenin3 dimer (Ejarque et al. 2013). Reporter assays show that ATOH8 lacks a transactivation domain and possesses intrinsic repressor activity that depends on a conserved Proline-rich domain (Ejarque et al. 2013). In zebrafish, ATOH8 is required during heart development and it genetically interacts with heart transcriptional regulators GATA4 and FOG1. Furthermore, it could form a protein complex with GATA4 and FOG2 in vitro (Rawnsley et al. 2013). During differentiation of hESC to endothelial cells, ATOH8 directly targets the promoter of an endothelial marker, eNOS, to regulate its expression (Fang et al. 2014). As an iron-regulation transcription factor (Kautz et al. 2008), ATOH8 is also shown to directly bind to the promoter of *Hmap*, which is involved in the maintenance of iron homeostasis (Patel et al. 2014).

PPP3CB is one of the three isoforms of the A subunit of calcineurin (CnA). Calcineurin is a calcium-dependent serine/threonine phosphatase. It consists of two subunits: subunit A (CnA) and subunit B (CnB). CnA comprises a catalytic domain followed by a B subunit regulatory site (Sikkink et al. 1995), a calmodulin binding site (Kincaid et al. 1988) and an inhibitory domain (Hashimoto et al. 1990). The last three regions constitute the regulatory region of CnA. Removal of the last two regions converts CnA to a constitutively active phosphatase independent of the calcium signal (Kim et al. 2009; Molkentin et al. 1998). The B subunit (CnB) is the regulatory subunit, which binds to calcium, stabilizes the protein complex and promotes the binding of calmodulin to CnA. Therefore as a mediator of calcium signaling, calcineurin translates the calcium signal into direct dephosphorylation of a number of target proteins including transcription factors, ion channels, apoptosis factors, cytoskeleton proteins and others (Li et al. 2011). For example, the family of transcription factors termed NFATs (nuclear factor of activated T cells) could be translocated from the cytoplasm into the nucleus to act as a functional transcription factors after dephosphorylation by calcineurin (Beals et al. 1997); phosphate groups of Tau protein have also been shown to be removed by calcineurin to prevent the aggregation of the protein in the brain (Gong et al. 1994).

For CnA, three isoforms PPP3CA (CnAα), PPP3CB (CnAβ) and PPP3CC (CnAγ) have been identified. Both α and β isoforms are expressed ubiquitously while γ is found to be present in testis and brain (Perrino et al. 2002). In spite of the overlapping expression of α and β isoforms in various tissues, it is proposed that they have distinct physiological roles as corroborated by various evidence: CnAα mutant mice have a smaller size and survive only a few weeks, while CnAβ mutant mice are able to live longer and are fertile (Gooch et al. 2004). CnAα plays exclusive roles in antigen-specific T cell response (Zhang et al. 1996) and kidney development (Gooch et al. 2004), while the β isoform is required for the T cell development and survival (Manicassamy et al. 2008). The distinct roles of CnAα and CnAβ in different tissues may be due to the differences in substrate selection (Perrino et al. 2002). In addition, the expression of the β isoform is specifically upregulated in the Alzheimer’s disease (AD) brain, suggesting a critical role of the β isoform during the pathogenesis of AD (Hata et al. 2001). Finally, the γ isoform was found to be associated with schizophrenia (Sacchetti et al. 2013).

In our study, we focus on the β isoform of calcineurin, PPP3CB, and demonstrate for the first time that ATOH8 interacts with PPP3CB and identify the regions of interaction in both proteins. While the conserved catalytic domain of calcineurin A is responsible for the direct interaction, the regulatory regions, especially the unique proline-repeats in the N-terminus of PPP3CB, seem to contribute to the selectivity of substrate partners, as PPP3CB but not PPP3CA has affinity to ATOH8 in spite of their conserved catalytic domain. Functionally, our results showed that calcineurin mediates ATOH8 nuclear translocation from cytoplasm, as ATOH8 remained in the cytoplasm after inhibiting calcineurin with CsA, revealing a potential new mechanism how the transcriptional activity of ATOH8 could be controlled by calcineurin-mediated calcium signaling.

## Results

### Primary structure of human ATOH8 and PPP3CB

**Fig. 1:**
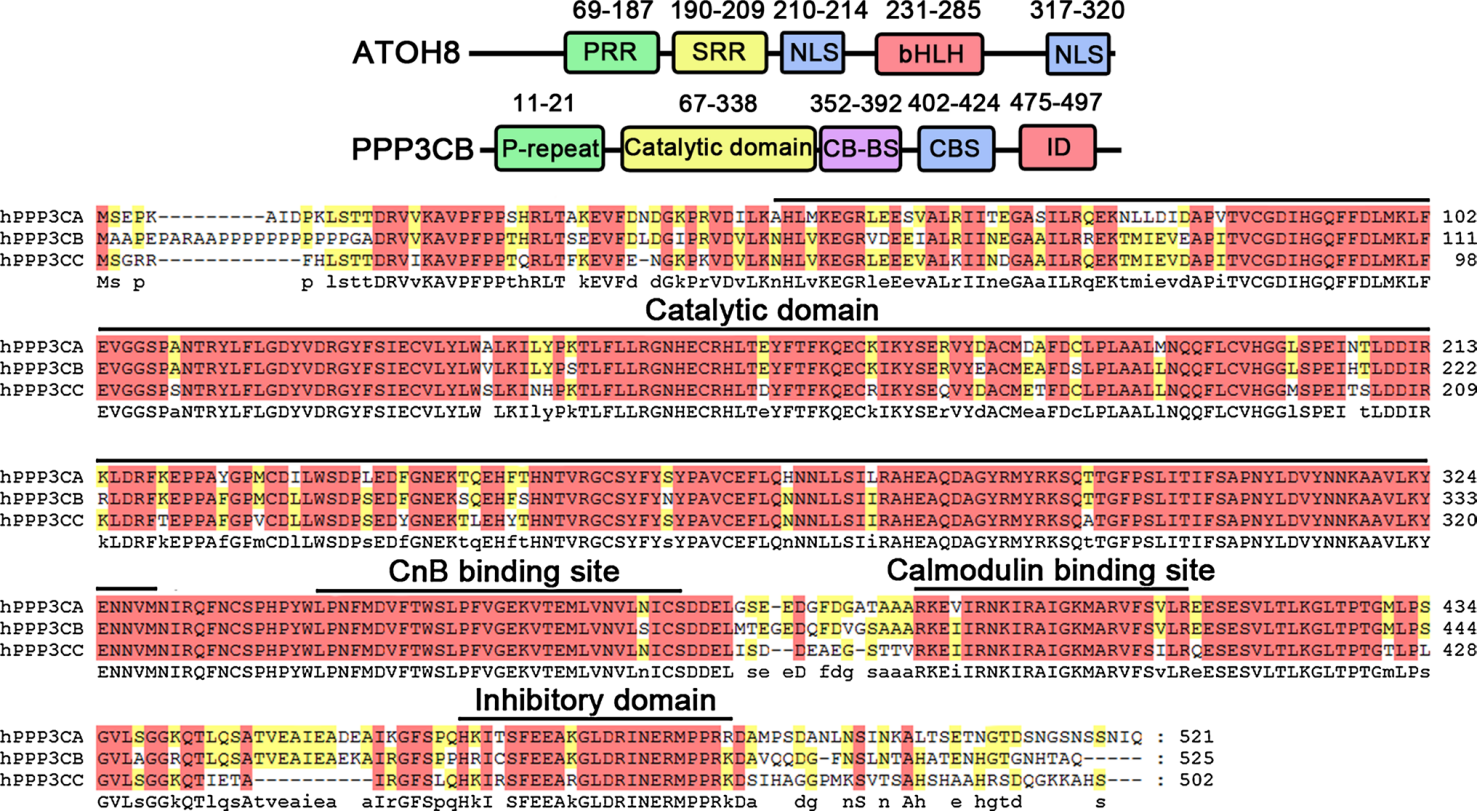
Primary structure of ATOH8 and PPP3CB. ATOH8 comprises a proline-rich region (PRR), a serine-rich region (SRR), a bHLH domain and two predicted nuclear localization signal (NLS). PPP3CB contains a proline repeat, a catalytic domain, a CnB binding site (CB-BS), a calmodulin binding site (CBS) and an inhibitory domain (ID). The phosphatase domain, CB-BS, CBS and ID are conserved among the three isoforms of CnA

Sequence analysis of human ATOH8 (NP_116216.2, Fig. 1) shows that there is a proline-rich region (PRR) ranging from residue 69 to 187. Within this region, 39 out of 119 residues are prolines. A serine-rich region (SRR) starts from residue 190 and ends at residue 209. It contains 9 serines out of the 20 residues. Adjacent to the C-terminus of the serine-rich region, a predicted nuclear localization signal (NLS; P_210_R_211_K_212_R_213_) is present. A bHLH domain spans from Arg231 to Leu285. The basic region of the bHLH domain consists of the residues from 231 to 242, while the HLH domain spans from 243 to 285. At the C-terminus of the protein locates another predicted NLS (K_317_K_318_R_319_K_320_).

Sequence alignment of PPP3CB with the other two isoforms PPP3CA and PPP3CC shows that all three isoforms have the conserved catalytic domain (phosphatase domain), a CnB binding site, a calmodulin binding site and an inhibitory domain (Fig. 1). In contrast to PPP3CA and PPP3CC, there is a proline repeat (from residue 11 to 21) unique to PPP3CB (Fig. 1).

### Interaction between PPP3CB and ATOH8

**Fig. 2:**
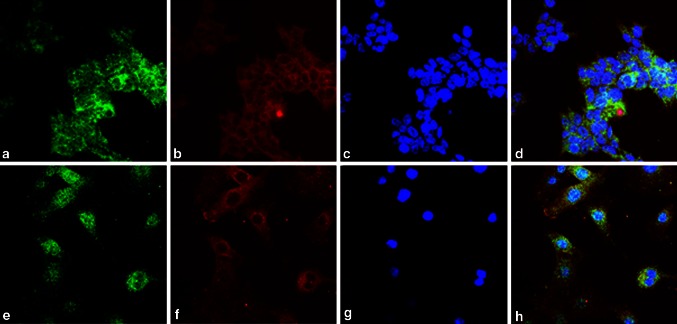
Co-localization of Atoh8 and PPP3CB in HEK293 and C2C12 cell lines. **a**–**d** In HEK293 cells, Atoh8 is shown in *green*, PPP3CB in *red*, and the nuclei were counterstained with DAPI in *blue*. **e**–**h** Shows the immunohistochemical stainings of ATOH8 (*green*) and PPP3CB (*red*) in C2C12 cells with the same antibodies used in HEK293 cells

**Fig. 3:**
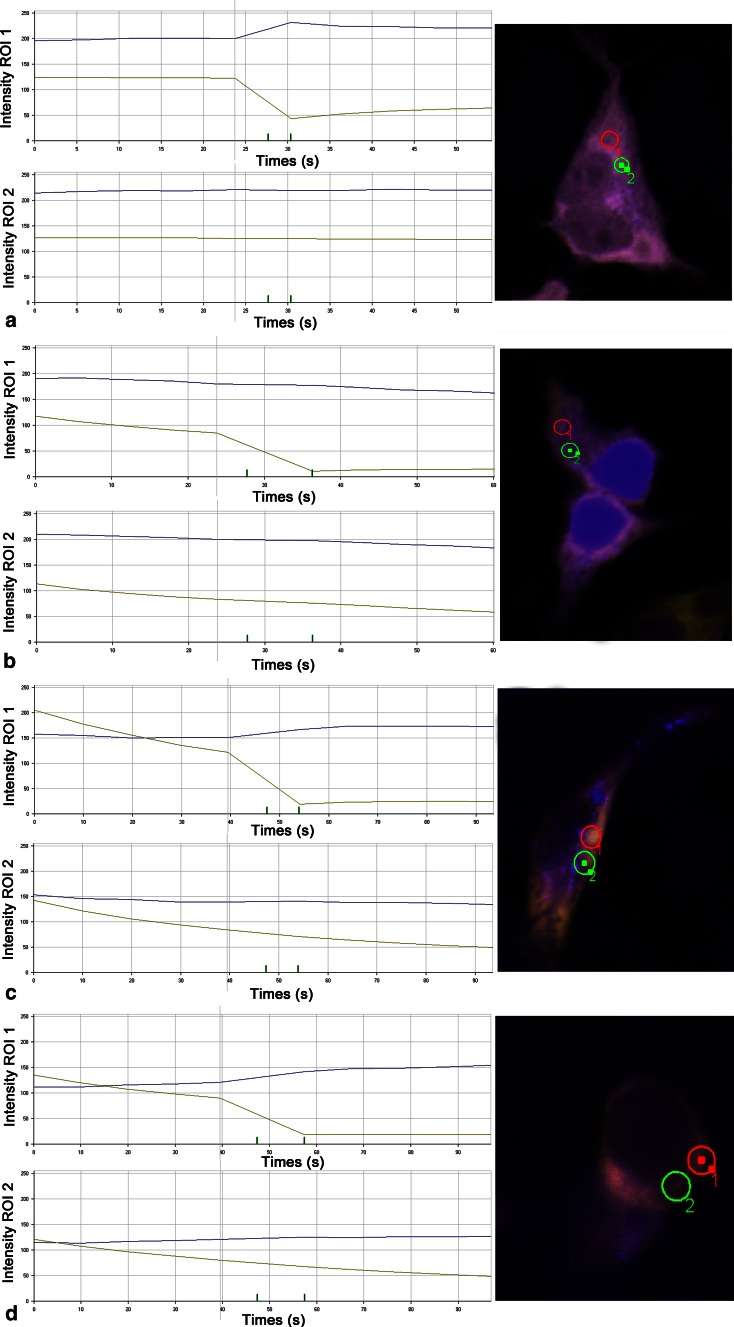
FRET between ATOH8-ECFP and EYFP-PPP3CB fusion protein. Region of interest 1 (ROI 1) is treated with YFP bleach. ROI 2 is the region without treatment of YFP bleach. **a** FRET of ECFP-EYFP fusion protein (positive control of FRET). In ROI 1, CFP intensity increases after bleaching YFP. In ROI 2, CFP intensity remains relatively stable. **b** No FRET between ECFP and EYFP-PPP3CB fusion protein (negative control of FRET). In both ROIs, CFP intensity remains relatively stable. **c** FRET between ATOH8-ECFP and EYFP-PPP3CB. CFP intensity increases after bleaching YFP as shown in ROI 1, while in ROI 2, CFP intensity remains relatively stable in ROI 2

Initially, the interaction between PPP3CB and ATOH8 was discovered from a yeast-2-hybrid screen (data not shown). To examine the possibility of their interaction in mammalian cells, we first investigated whether they co-localize or not in two different cell lines, HEK293 and C2C12. The results showed that they indeed co-localized in the cytoplasm of the cells from these two lines (Fig. 2). To confirm the interaction, we performed fluorescence resonance energy transfer (FRET) experiments. We ectopically expressed ECFP-EYFP fusion protein in HEK293 cells as positive control, ECFP and EYFP-PPP3CB fusion protein as negative control, and ATOH8-ECFP fusion protein paired with EYFP-PPP3CB fusion protein for FRET examination. We selected those cells where ECFP and EYFP co-localize in the cytoplasm for FRET analysis. The results showed that for both positive control and the pair (ATOH8-ECFP and EYFP-PPP3CB), FRET occurred in the examined region of interest (ROI), as CFP intensity increased after bleaching YFP (Fig. 3a, c). For negative control, CFP intensity decreased after bleaching YFP in the examined ROI (Fig. 3b).

**Fig. 4:**
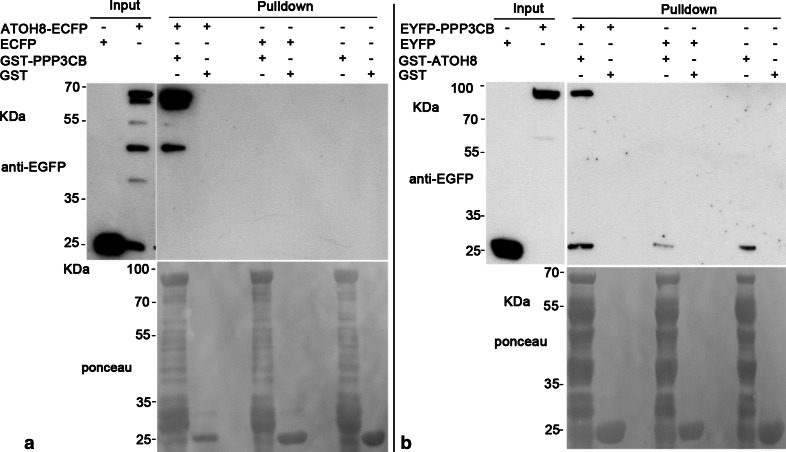
Pulldown between ATOH8 and PPP3CB. **a** GST-PPP3CB pulls down ATOH8-ECFP. GST-PPP3CB (~87KDa) and GST (~26 kDa, control) are the baits. Lysates containing ATOH8-ECFP (~61 kDa) and ECFP (~25 kDa, control) are preys. ATOH8-ECFP is specifically pulled down by GST-PPP3CB. **b** GST-ATOH8 pulls down EYFP-PPP3CB. GST-ATOH8 (~61 kDa) and GST (~26 kDa, control) are the baits. Lysates containing EYFP-PPP3CB (~87 kDa) and EYFP (~25 kDa, control) are preys. EYFP-PPP3CB is specifically pulled down by GST-ATOH8

The interaction between ATOH8 and PPP3CB could be corroborated by our pulldown experiments. We enriched the GST-PPP3CB fusion protein and incubated GST-PPP3CB with mammalian cell lysates containing overexpressed ATOH8-ECFP fusion protein. The results showed that GST-PPP3CB fusion protein (~87KDa) pulled down ATOH8-ECFP (~61KDa), while it did not show any affinity to ECFP (Fig. 4a). Furthermore, GST alone did not pull down either ATOH8-ECFP or ECFP (Fig. 4a). Consistently, the GST-ATOH8 fusion protein (~61KDa) pulled down EYFP-PPP3CB (~ 87KDa) as well (Fig. 4b).

**Fig. 5:**
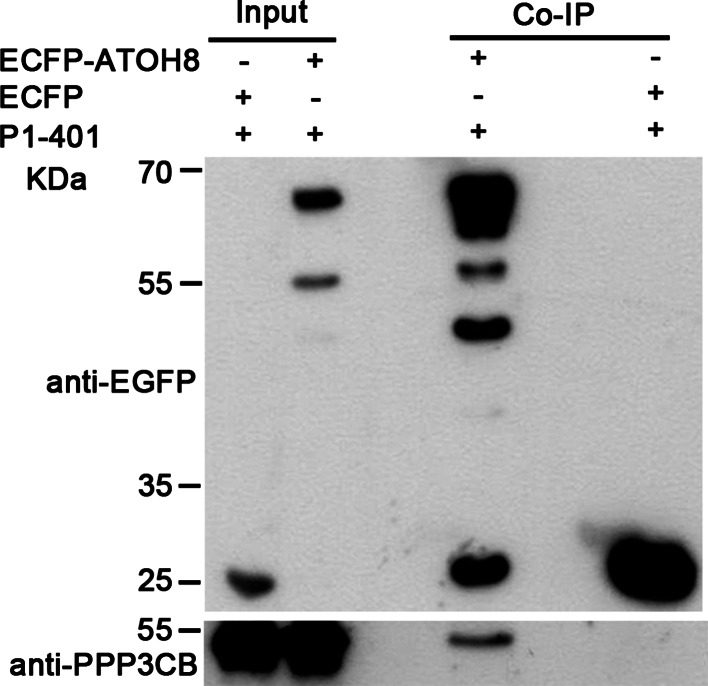
Co-immunoprecipitation of ATOH8 and active PPP3CB (P1-401). P1-401 (~47 kDa) is co-immunoprecipitated with ATOH8-ECFP (~61 kDa)

To further verify this interaction in mammalian cells, we co-expressed constitutively active PPP3CB (referred to as P1-401) and ATOH8-ECFP fusion protein in mammalian cells and performed co-immunoprecipitation (Co-IP). The results show that ATOH8-ECFP fusion protein is able to precipitate the active PPP3CB (~47 kDa; Fig. 5), but not the full-length PPP3CB (data not shown).

**Fig. 6:**
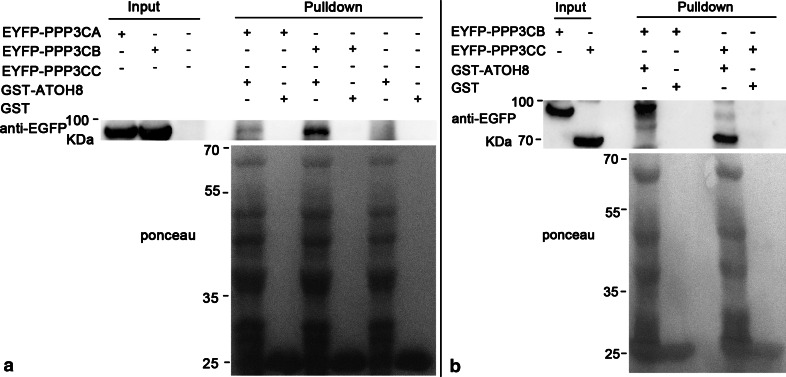
Full-length PPP3CA shows no affinity to ATOH8. **a** EYFP-PPP3CA expressed in Cos7 cells could not be pulled down by GST-ATOH8, in contrast to the positive control EYFP-PPP3CB. Full-length EYFP-PPP3CC could not be detected in the input. **b** Truncated PPP3CC interacts with ATOH8. In HEK293 cells, no full-length EYFP-PPP3CC is detected. Instead, the truncated EYFP-PPP3CC is produced and pulled down by GST-ATOH8. The truncated EYFP-PPP3CC has a molecular weight of about 70 kDa. Minus the ~27 kDa of EYFP, the remaining peptide has a molecular weight of ~43 kDa, which is close to that of the catalytic domain of PPP3CC

While the interaction between PPP3CB and ATOH8 was confirmed, we were also interested to know whether the other two isoforms of calcineurin A, PPP3CA and PPP3CC, interact with ATOH8 or not. Our pulldown assay showed that there was no affinity between full-length PPP3CA and ATOH8 (Fig. 6a). We used isoform 2 (NM_005605.4) of PPP3CC for the pulldown experiment and observed that overexpression of this isoform caused severe death of Cos7 cells and produced a truncated protein (data not shown). We then tried the experiment in HEK293T cells. Again, cell death was observed (data not shown) and only a truncated protein of PPP3CC was produced which showed interaction with ATOH8 (Fig. 6b).

In summary, our results from different methods provide sufficient evidence that ATOH8 interacts with PPP3CB, but not with PPP3CA, while the interaction with PPP3CC needs to be further investigated.

### Mapping the regions of interaction of ATOH8 and PPP3CB

**Fig. 7:**
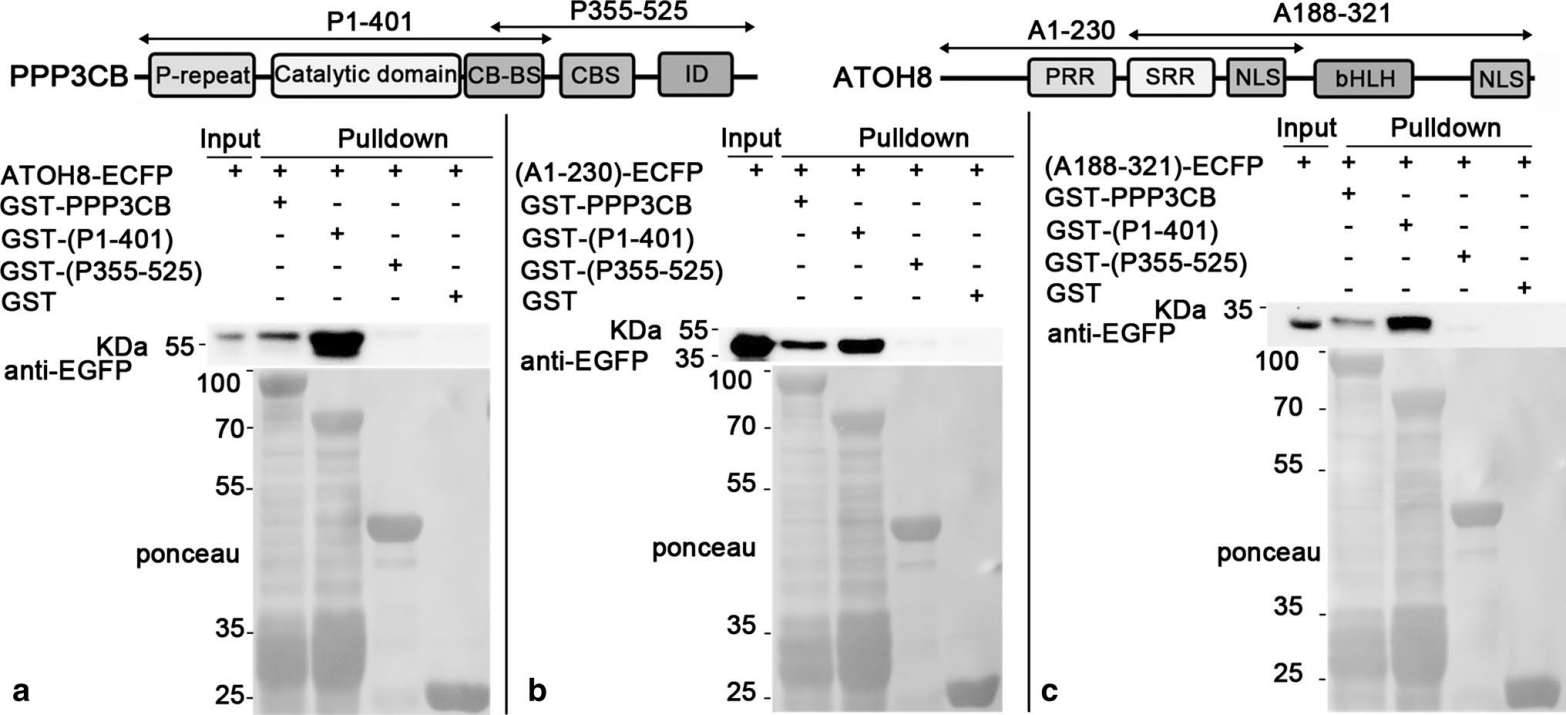
P1-401 pulls down ATOH8. **a** GST-PPP3CB (~87 kDa) and GST-(P1-401; ~72 kDa) pull down ATOH8-ECFP (~61 kDa). **b** GST-PPP3CB and GST-(P1-401) pull down (A1-230)-ECFP (~52 kDa). **c** GST-PPP3CB and GST-(P1-401) pull down (A188-321)-ECFP (~43 kDa)

To dissect which regions of the two proteins that are responsible for the interaction, we first split ATOH8 into two parts (A1-230 and A188-321, respectively, Fig. 7) and fused them with ECFP, respectively. PPP3CB was divided into two parts as well (P1-401 and 355–525, respectively, Fig. 7), which were fused with GST. Using the GST fusion proteins as baits, we performed pulldown assay again. The results showed that both GST-PPP3CB (~87 kDa) and GST-(P1-401) (~72 kDa) pulled down ATOH8-ECFP (~61 kDa, Fig. 7a), (A1-230)-ECFP (~52 kDa, Fig. 7b) and (A188-321)-ECFP (~43 kDa, Fig. 7c). GST-(P355-525) did not pull down any prey protein (Fig. 7). In addition, it seemed that P1-401 had a stronger affinity to ATOH8 than the full-length PPP3CB, as shown by more intensive bands detected by EGFP antibody (Fig. 7).

**Fig. 8:**
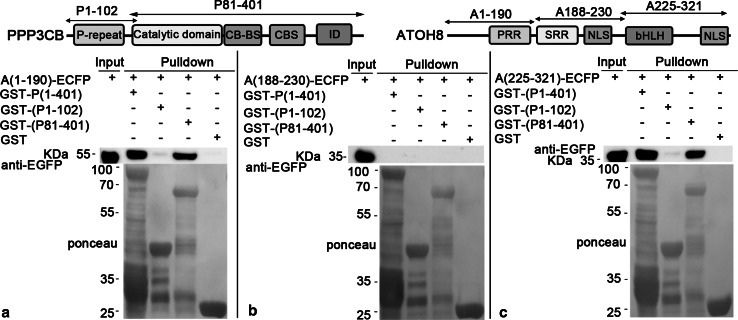
P81-401 pulls down A1-190 and A225-321. **a** GST-(P1-401) (~72 kDa) and GST-(P81-401) (~64 kDa) pull down (A1-190)-ECFP (~48 kDa). **b** (A188-230)-ECFP (~32 kDa) is not pulled down by any bait protein. **c** GST-(P1-401; ~72 kDa) and GST-(P81-401; ~64 kDa) pull down (A25-321)-ECFP (~39 kDa)

To further analyze the interaction regions, we divided P1-401 into two parts, P1-102 and P81-401, and fused them with GST, respectively. As both A1-230 and A188-232 showed affinity with PPP3CB, we thus split ATOH8 into A1-190, A118-230 and A225-321 and fused them with ECFP. Our data clearly show that GST-(P81-401) pulled down both A1-190 (~48 kDa, Fig. 8a) and A225-321 (~39 kDa, Fig. 8c), but not the A118-230 (Fig. 8b).

**Fig. 9:**
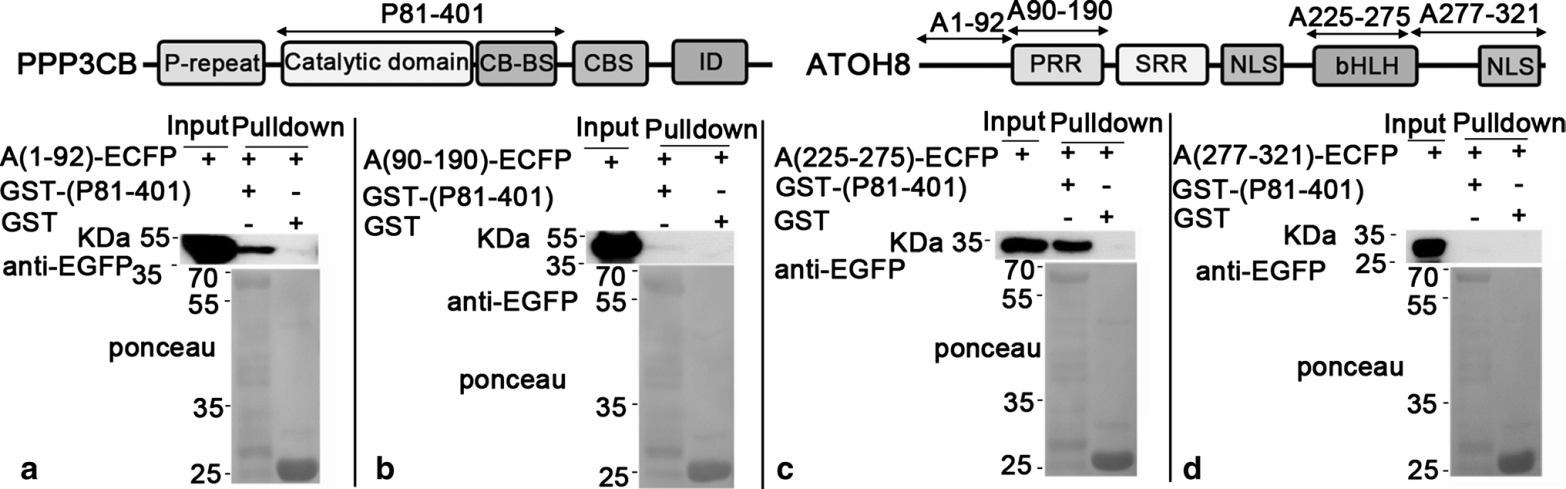
A1-92 and A225-275 are pulled down by P81-401. **a**, **c** P81-401 pulls down (A1-92)-ECFP (~38 kDa) and (A225-275)-ECFP (~34 kDa). **b**, **d** (A90-190)-ECFP (~38 kDa) and (A277-321)-ECFP (~33 kDa) are not pulled down by any bait

**Fig. 10:**
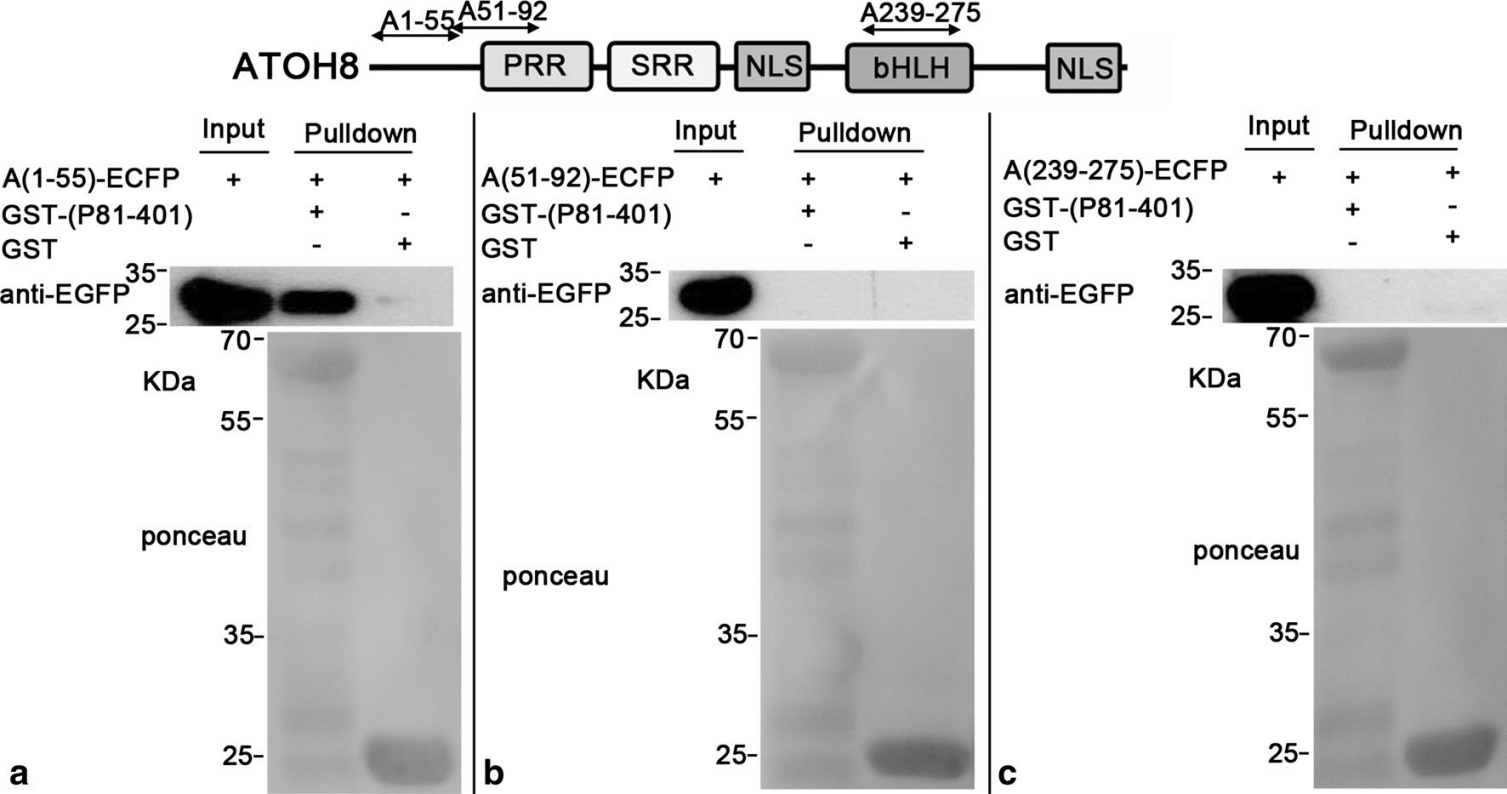
A1-55 is pulled down by P81-401. **a** P81-401 pulls down (A1-55)-ECFP (~32 kDa) and (A225-275)-ECFP. **b**, **c** A51-91 and A239-275 have no affinity to P81-401

Subsequent pulldown experiments showed that P81-401 pulled down A1-92 (~38 kDa, Fig. 9a) and A225-275 (~34 kDa, Fig. 9d), while A90-190 and A255-321 were not pulled down by P81-401 (Fig. 9b, c). Moreover, P81-401 pulled down A1-55 (~34 kDa, Fig. 10a) rather than A51-92 (~32 kDa, Fig. 10b). In contrast, the shortened A225-275 (A239-275) lost the affinity to P81-401 (Fig. 10c).

To sum up, we have narrowed down the interaction regions of both proteins with pulldown assays: For PPP3CB, it ranges from residues 81–401 (P81-401), which contains the phosphatase domain; for ATOH8, two separate interaction regions have been found. One is located in the N-terminus of ATOH8 (A1-55), and the other is within the bHLH domain (A225-275).

### Inhibiting calcineurin prevents nuclear accumulation of ATOH8

**Fig. 11:**
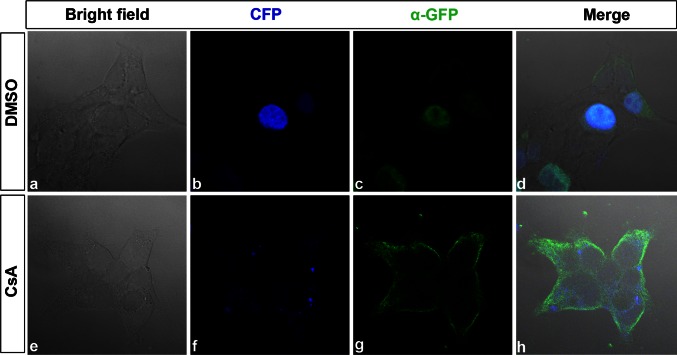
ATOH8-ECFP fusion protein is retained in the cytoplasm. **a**–**d** In DMSO-treated HEK293 cells, ATOH8-ECFP fusion protein is majorly localized in the nucleus as shown by CFP (**b**) and GFP antibody staining (**c**). **e**–**h** After treatment with 10 μM CsA, ATOH8-ECFP is retained in the cytoplasm

After confirming the interaction between ATOH8 and PPP3CB, we were interested to know the physiological role of this interaction. We hypothesized that PPP3CB may affect the subcellular localization of ATOH8 by dephosphorylating ATOH8, probably in a way similar to the nuclear localization of NFATs mediated by calcineurin. To validate the hypothesis, we inhibited calcineurin with cyclosporin A (CsA), a calcineurin inhibitor, in HEK293 cells after ectopically expressing ATOH8. The results showed that the ectopically expressed fusion protein ATOH8-ECFP was retained in the cytoplasm after CsA treatment (Fig. 11e–h). In contrast, ATOH8-ECFP was majorly localized in the nuclei with DMSO treatment (Fig. 11a–d).

## Discussion

ATOH8 is a bHLH transcription factor expressed in a variety of different tissues such as brain, heart, skeletal muscle, kidney and pancreas (Lynn et al. 2008; Wang et al. 2015). Functional analyses have revealed that it regulates the differentiation of neural precursors (Inoue et al. 2001), pancreas precursor cells (Lynn et al. 2008) and podocytes (Ross et al. 2006) and the development of retina and muscles (Balakrishnan-Renuka et al. 2014; Yao et al. 2010), and other tissues. To function as a transcription factor, the nuclear localization of the protein is the key in order to bind to the target DNA. As seen from the structure of ATOH8, the protein comprises two predicted sites of NLS, and one of them is adjacent with the serine-rich region (SRR). This SRR is liable to phosphorylation and dephosphorylation which may either mask or expose the neighboring NLS. As a result, the cytoplasmic protein could be retained in the cytoplasm or shuttled into the nucleus. From our results, we have shown that the catalytic subunit of calcineurin, PPP3CB, interacts with ATOH8. Assumedly, as a serine/threonine-dependent phosphatase, calcineurin may dephosphorylate ATOH8 and thus affects its subcellular localization. Indeed, we found that ATOH8 nuclear localization was blocked after inhibiting calcineurin with CsA. This is reminiscent of NFAT nuclear translocation from the cytoplasm (Okamura et al. 2000; Beals et al. 1997). In the cytoplasm, SRR of NFAT is initially phosphorylated which masks the NLS of the protein. Upon the activation of calcineurin, it dephosphorylates the SRR and exposes the NLS, leading to the translocation of NFAT from the cytoplasm to the nucleus (Okamura et al. 2000). It is highly possible that the subcellular localization of ATOH8 is mediated in a similar way by calcineurin, but the individual steps remain to be investigated.

The interaction domain of PPP3CB, which is almost equivalent to the phosphatase domain, is conserved among the three different isoforms of calcineurin A. However, our results showed that ATOH8 interacts with PPP3CB, but not with PPP3CA. This may be due to the distinct substrate preferences of the isoforms in spite of their almost identical catalytical and regulatory domains (Perrino et al. 2002). Furthermore, it has been reported that the proline-rich region, which is uniquely present in PPP3CB, is involved in specific substrate selection because the affinity of PPP3CB to its substrates dramatically decreases after removal of the proline-rich region (Kilka et al. 2009). These may explain the affinity of ATOH8 to PPP3CB instead of PPP3CA.

At least two different regions of ATOH8 were identified to interact with PPP3CB. Previous studies showed that two conserved motives PxIxIT and LxVP mediate the interaction between CnA and its substrate proteins (Li et al. 2011). We did not find such motives in these two regions of ATOH8, suggesting that proteins without the two motives could also interact with CnA. In addition, one of the interaction regions is located in the bHLH domain. As shown by our previous study, bHLH domain of ATOH8 orthologous is highly conserved among metazoans (Chen et al. 2011). And the structure of PPP3CB, especially the catalytic domain, is also highly conserved in metazoans. Thus the interaction of ATOH8 and PPP3CB mediated by the two conserved domains could be conserved throughout evolution as well. However, another region of interaction is located in the N-terminal end of ATOH8, which is less conserved among vertebrates (Chen et al. 2011). This interaction region may emerge as a regulatory region during evolution and could be responsible for the regulation of extra affinity of ATOH8 to PPP3CB.

In summary, we have provided sufficient evidence of a novel interaction between ATOH8 and calcineurin and identified the regions which are responsible for the interaction. Our results further show that calcineurin mediates the subcellular localization of ATOH8, which reveals the regulation of ATOH8 transcriptional activity by calcium signaling via calcineurin.

## Materials and methods

### Plasmids

The plasmid constructs for the study were listed in Supplementary Table 1. Most of the plasmids were constructed in the PCR-based way. The primers, vectors and cloning sites selected were shown in Supplementary Table 1. ATOH8 (NM_032827.6) cDNA clone was from our previous study (Chen et al. 2011). PPP3CB (NM_001142353.2) cDNA clone was purchased (Origene). PPP3CA (NM_000944.4) cDNA clone was from Addgene (Addgene plasmid 11787); cDNA of PPP3CC isoform 2 (NM_005605.4) was cloned by RT-PCR. All constructs were verified by Sanger sequencing.

### Antibodies

The primary antibodies used in this study included ATOH8 antibody (Sigma, AV 39728), PPP3CB antibody (Santa Cruz, sc-6124), EGFP antibody (Invitrogen, A11122) and GFP antibody JL-8 (Clontech, 632380). The secondary antibodies, anti-rabbit (Alexa fluor 488) and anti-mouse (Alexa fluor 568 and Alexa fluor 488), were used for immunostaining of HEK293 and C2C12 cell, while HRP-linked anti-rabbit (Sigma, G21234), HRP-linked anti-goat (Santa Cruz, SC-2020) were used for Western blot. DAPI (invitrogen, D1306) was used for nucleus counterstaining.

### Cell culture and transfection

HEK293, Cos7 and C2C12 cells were cultured in Dulbecco’s modified Eagle’s medium (DMEM) with high glucose containing 2 mM glutamine supplemented with 10 % FCS and 1 % penicillin–streptomycin in the incubator of 37 °C and 5 % CO_2_. HEK 293 cells were transfected with polyethylenimine (PEI) (Polyplus) or MATra (IBA). Cos7 cells were transfected with PolyFect (Qiagen). Cells were fixed with 4 % PFA/PBS or lysed 48 h after transfection.

### Fluorescence resonance energy transfer (FRET)

HEK293T cells were cultured on coverslips. Plasmids pATOH8-ECFP and pEYFP-PPP3CB were co-transfected into HEK293T cells. pECFP-N1 and pEYFP-PPP3CB were co-transfected as negative control, respectively, pECFP-EYFP as positive control. 48 h after transfection, cells were fixed in 4 % PFA/PBS for 30 min and washed with PBS. Acceptor photobleach method was used to detect FRET under laser confocal scanning microscope (LSM 510, Zeiss) associated with AIMS software. Firstly 405 and 515 nm wavelength of laser was used to scan cells to select both ECFP and EYFP positive cells. In the selected regions of interest, YFP was bleached at 514-nm wavelength with ten times iterations. Before and after bleaching YFP, regions of interest (ROIs) were scanned every 2 s at both wavelengths for five times and the real-time intensities of CFP and YFP in the ROI were recorded automatically by the software in the time window.

### Pulldown

GST fusion protein was expressed in BL21 cells and purified with glutathione beads (Macherey–Nagel) following the manual. ECFP or EYFP fusion protein was expressed in Cos7 or HEK293T cells, and cell lysate was collected to incubate with glutathione beads bound with GST fusion protein for 2–4 h at 4 °C on a rotator. Beads were washed with lysis buffer for 3–5 times. The protein bound by beads was eluted with 50–100 µl 2× Laemmli buffer at 95 °C for 5–10 min and followed by SDS-PAGE and Western blot.

### Co-immunoprecipitation

Plasmid pairs, pECFP-ATOH8 and pIRES2-dsRed2, pECFP-ATOH8 and pIRES2-dsRed2-(P1-401), were co-transfected into COS7 cells, respectively. 48 h after transfection, cells were lysated with lysis buffer (PBS + 1 % Triton 100 + 1× protease inhibitor, 250 mM NaF, 10 mM Na_3_VO_4_). Lysates were incubated with GFP-trap_A beads (Chromotek) for 2 h at 4 °C. Beads were washed with lysis buffer for three times. Protein bound with beads was eluted with 2× Laemmli buffer and followed by 10 % SDS-PAGE and Western blot.

### Western blot

Protein samples were boiled in 1× Laemmli buffer at 95 °C for 5 min, separated by 10 % SDS-PAGE gel and blotted onto the nitrocellulose membrane. Blotted membranes were incubated with proper primary antibodies overnight. The membrane was washed with 1× PBST for three times and incubated with HRP-conjugated secondary antibodies for 2 h at room temperature. Finally, the protein was detected by SuperSignal West Pico Chemiluminescent Substrate (Thermo Scientific, 34077).

### Cyclosporin A treatment

HEK293 cells were transfected with pATOH8-ECFP plasmid with MATra (IBA). 24 h after transfection, cells were treated with 10 µM CsA (Sigma, 30024-25MG) for 24 h. For the control, transfected cells were cultured with 0.1 % DMSO. After the treatment, the cells were fixed and subjected to immunostaining with JL-8 antibody (Clontech, 632380) to detect ECFP. All experiments were repeated three times.

## Electronic supplementary material

Supplementary material 1 (DOC 54 kb)
