# Supplementary material for: A novel interaction between ATOH8 and PPP3CB
Source: Histochem Cell Biol. 2015 Oct 26;145:5–16. doi: 10.1007/s00418-015-1368-5 (PMC4710663; doi:10.1007/s00418-015-1368-5)
Supplement: Supplementary file 1 — Supplementary material 1 (DOC 54 kb) [file 418_2015_1368_MOESM1_ESM.doc]

**Supplementary Table 1. Plasmids list. The table shows the information of vectors, primers and cloning sites for the plasmid constructs used in this study.**

| **Plasmids** | **Vectors** | **Forward primers** | **Reverse primers** | **cloning sites** |
| --- | --- | --- | --- | --- |
| pEYFP-ECFP | pECFP-N1 (clontech) | TCAGATCCGCTAGCGCTA | GTCTAGAGAAGCTTCTTGTACAGCTCGTCCATGCCG | *Hind III, NheI* |
| pATOH8-ECFP | pECFP-N1 (clontech) | GATGGCTGAAGCTTGCCACCATGAAGCACATCCCGGT | GTCTAGGGATCCAGCTCCTTGCGCTTCTTG | *Hind III, BamHI* |
| pEYFP-PPP3CA | pEYFP-Tub (clontech) | GAGCTGCTCGAGTGATGTCCGAGCCCAAGGC | GTCTAGAGGGATCCTCACTGAATATTGCTGCTATTACTGC | *XhoI, BamHI* |
| pEYFP-PPP3CB | pEYFP-Tub (clontech) | GAGCTGCTCGAGTGATGGCCGCCCCGGAGCCGGCCC | GTCTAGAGGGATCCTCACTGGGCAGTATGGTTGC | *XhoI, BamHI* |
| pEYFP-PPP3CC | pEYFP-Tub (clontech) | GAGCTGCTCGAGTG ATGTCCGGGAGGCGCTTC | GTCTAGAGCTCGAGTGTCATGAATGGGCTTTCTTCCC | *XhoI, BamHI* |
| Constitutively active PPP3CB | pEYFP-PPP3CB | *MfeI* digestion removes the regulatory regions of PPP3CB | | |
| pGST-ATOH8 | pGEX-6P-1 (GE Healthcare) | *GAGCTGCT*GGATCCatgaagcacatcccggtc | GTCTAGAGAATTCtcactccttgcgcttcttg | *BamHI, EcoRI* |
| pGST-PPP3CB | pGEX-6P-1 | GAGCTGCTggatccATGGCCGCCCCGGAGCCGGCCC | GTCTAGAGgaattcTCACTGGGCAGTATGGTTGC | *BamHI, EcoRI* |
| pGST-P1-401 | pGEX-6P-1 | GAGCTGCTggatccATGGCCGCCCCGGAGCCGGCCC | cgGAATTCTCAggctgcagctgaacctacatc | *BamHI, EcoRI* |
| pGST-(P355-525) | pGEX-6P-1 | CGggatcctttatggatgtcttcacgtggt | GTCTAGAGgaattcTCACTGGGCAGTATGGTTGC | *BamHI, EcoRI* |
| pGST-(P1-102) | pGEX-6P-1 | GAGCTGCTggatccATGGCCGCCCCGGAGCCGGCCC | cgGAATTCTCAgccatggatgtcaccacac | *BamHI, EcoRI* |
| pGST-(P81-401) | pGEX-6P-1 | CGggatccCTTCGGAGAGAGAAAACCATG | cgGAATTCTCAggctgcagctgaacctacatc | *BamHI, EcoRI* |
| p(A1-230)-ECFP | pECFP-N1 (clontech) | GATGGCTGAAGCTTGCCACCATGAAGCACATCCCGGT | CGGGATCCAGggtctgctgcagggcttt | *Hind III, BamHI* |
| p(A188-321)-ECFP | pECFP-N1 | GTGCTGAAGCTTGCCACCATGggggaaagttcctactcgtca | GTCTAGGGATCCAGCTCCTTGCGCTTCTTG | *Hind III, BamHI* |
| p(A1-190)-ECFP | pECFP-N1 | GATGGCTGAAGCTTGCCACCATGAAGCACATCCCGGT | CGGGATCCAGactttccccggggcgc | *Hind III, BamHI* |
| p(A188-230)-ECFP | pECFP-N1 | GTGCTGAAGCTTGCCACCATGggggaaagttcctactcgtca | CGGGATCCAGggtctgctgcagggcttt | *Hind III, BamHI* |
| p(A225-321)-ECFP | pECFP-N1 | GTGCTGAAGCTTGCCACCatgAAAGCCCTGCAGCAGACC | GTCTAGGGATCCAGCTCCTTGCGCTTCTTG | *Hind III, BamHI* |
| p(A1-92)-ECFP | pECFP-N1 | GATGGCTGAAGCTTGCCACCATGAAGCACATCCCGGT | CGGGATCCgtgtccgtgccccctc | *Hind III, BamHI* |
| p(A90-190)-ECFP | pECFP-N1 | GTGCTGAAGCTTGCCACCATGacggacacagccgggg | CGGGATCCAGactttccccggggcgc | *Hind III, BamHI* |
| p(A225-275)-ECFP | pECFP-N1 | GTGCTGAAGCTTGCCACCatgAAAGCCCTGCAGCAGACC | CGGGATCCcaggcgatcctcaggatg | *Hind III, BamHI* |
| p(A277-321)-ECFP | pECFP-N1 | GTGCTGAAGCTTGCCACCatgtacatcctgtccctggcg | GTCTAGGGATCCAGCTCCTTGCGCTTCTT | *Hind III, BamHI* |
| p(A1-55)-ECFP | pECFP-N1 | GATGGCTGAAGCTTGCCACCATGAAGCACATCCCGGT | CGGGATCCgctacggcgcggggctc | *Hind III, BamHI* |
| p(A51-92)-ECFP | pECFP-N1 | GTGCTGAAGCTTGCCACCATGgagccccgcgccgtag | CGGGATCCgtgtccgtgccccctc | *Hind III, BamHI* |
| p(A239-275)-ECFP | pECFP-N1 | GTGCTGAAGCTTGCCACCatggccagggagcggacgc | CGGGATCCcaggcgatcctcaggatg | *Hind III, BamHI* |
| pIRES2-dsRed2-P(1-401) | pIRES2-dsRed2 (Clontech) | GAGCTGCTCGAGTGATGGCCGCCCCGGAGCCGGCCC | GTCTAGAGgaattcTCACTGGGCAGTATGGTTGC | *XhoI, EcoRI* |
